# Two-Dimensional Shear Wave Elastography Evaluation of Post-transplantation Complications in Pediatric Receipt: A Retrospective Cohort

**DOI:** 10.3389/fped.2022.918145

**Published:** 2022-07-27

**Authors:** Li-hong Gu, Zi-cheng Lv, Hao-xiang Wu, Yu-Chen Hou, Run-lin Gao, Zhi-feng Xi, Hua Fang, Hao Feng, Li-xin Jiang, Qiang Xia

**Affiliations:** ^1^Department of Liver Surgery, Renji Hospital, School of Medicine, Shanghai Jiao Tong University, Shanghai, China; ^2^Department of Ultrasound, Renji Hospital, School of Medicine, Shanghai Jiao Tong University, Shanghai, China; ^3^Department of Pathology, Renji Hospital, School of Medicine, Shanghai Jiao Tong University, Shanghai, China; ^4^Shanghai Engineering Research Centre of Transplantation and Immunology, Shanghai, China; ^5^Shanghai Institute of Transplantation, Shanghai, China

**Keywords:** transplantation, two-dimensional shear wave elastography, ultrasound, pediatric, complications (COMP)

## Abstract

**Background:**

The 20-year survival rate in pediatric patients after liver transplantation (LT) was no more than 70%. Hepatic fibrosis is one of the principal factors affecting the long-term prognosis. Imaging evaluation was the first-line examination for pediatric liver graft assessment. However, the sensitivity and specificity were insufficient. Thus, two-dimensional shear wave elastography (2D-SWE) was performed to evaluate liver graft stiffness and complication in post-transplant pediatric receipt.

**Materials and Methods:**

In this retrospective cohort, 343 pediatric recipients who underwent liver graft biopsy in our tertiary LT center were recruited between June 2018 and December 2020. The 2D-SWE evaluation, laboratory examination, routine post-transplant biopsy, and hepatic pathological assessment were performed.

**Results:**

Ninety-eight of the 343 pediatric patients were included according to the protocol. The Liver Stiffness Measurements (LSM) value of 2D-SWE was significantly elevated in post-transplant fibrosis (*p* < 0.0001). The LSM value of patients with post-transplant biliary complications (*p* < 0.0001) and biopsy-proven rejection (BPR, *p* = 0.0016) also rose compared to regular recovery patients. Concerning the sensitivity and specificity of 2D-SWE in diagnosing liver graft fibrosis, the area under the ROC curve (AUC) was 88%, and the optimal cutoff value was 10.3 kPa.

**Conclusion:**

Pediatric LSM by 2D-SWE was efficient. Routine 2D-SWE evaluation could be optimal to predict significant liver graft fibrosis.

## Highlights

- This article provides insight into the effects of Two-dimensional shear wave elastography (2D-SWE) and the Liver Stiffness Measurements (LSM) on pediatric liver graft assessment.
- Liver Stiffness Measurements value of 2D-SWE was elevated in post-transplant fibrosis, post-transplant biliary complication, and biopsy-proven rejection.

## Introduction

Liver transplantation (LT) is the most effective treatment of end-stage liver disease in pediatric patients. However, the 5 and 10-year survival rates in pediatric patients after LT were 83 and 80%, respectively, and the 20-year survival rate was no more than 70% (1–3). The principal factors affecting the long-term prognosis in pediatric receipt contained viral infection (Epstein-Barr virus, cytomegalovirus), liver graft rejection, hepatic fibrosis, biliary tract complications, and drug-induced injure (4–7). Though liver biopsy is the golden standard for assessing liver disease and abnormal liver function, it required sedation for pediatric patients and induced approximately 20% post-biopsy discomfort and pain (8, 9). Therefore, a liver biopsy might not be the optimal approach for monitoring the post-transplant chronic rejection and liver injury in pediatric patients.

Imaging evaluation, including ultrasound, was the first-line examination for liver graft assessment, which was considered non-invasive and non-radiation, especially for children. However, the sensitivity and specificity were insufficient (10). By contrast, pediatric patients may not be able to hold their breath long enough to complete the computed tomography (CT) and magnetic resonance imaging (MRI) scan; infantile patients usually require sedation to maintain high-quality CT/MRI imaging. Routine ultrasound examination is requisite in post-transplant pediatric patients to evaluate the morphology of the allograft and macrovascular patency. However, conventional ultrasound is not diagnostic, especially for the earlier stages of fibrosis (11). Two-dimensional shear wave elastography (2D SWE) is a non-invasive technique for measuring liver stiffness (12, 13). Distinguished from transient elastography (TE), 2D-SWE was equipped with conventional ultrasound equipment, displaying both real-time gray scale ultrasound images for assessing morphologic changes and real-time quantitative map without stress concentration artifacts for assessing liver stiffness. Though some literature had reported the utility of 2D-SWE in evaluating liver fibrosis in hepatitis B, hepatitis C, or non-alcoholic fatty liver disease (NAFLD) (14, 15), little is known about the assessment of 2D-SWE in liver graft after pediatric LT.

In this study, 2D-SWE was performed to evaluate liver graft stiffness in post-transplant pediatric patients. The results were compared with pathologic findings of liver graft biopsy to clarify the value of 2D-SWE for pediatric patients.

## Materials and Methods

### Study Participants

**FIGURE 1:**
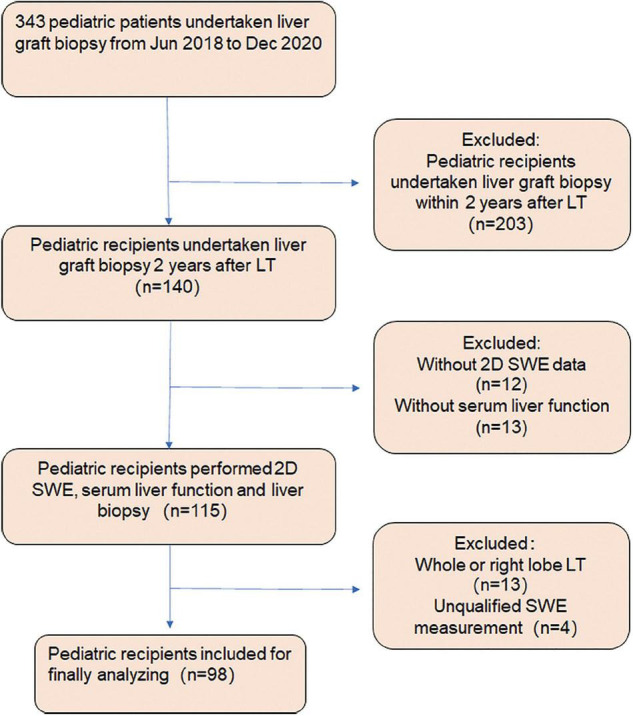
Flowchart of the study design protocol. Three hundred forty-three pediatric patients underwent liver graft biopsy from June 2018 to December 2020. One hundred and forty pediatric patients received liver biopsy within 2 years after liver transplantation (LT). The pediatric patients without 2D SWE data (*n* = 12) or serum liver function (*n* = 13), with whole liver or right lobe transplantation (*n* = 13) and unsuccessful 2D SWE examination (*n* = 4), were excluded from the study. Ninety-eight pediatric patients were recruited, including 86 subjects with left lateral lobe transplantation and 12 with left lobe graft.

In this retrospective cohort study, 343 pediatric patients underwent liver graft biopsy in our tertiary liver transplantation department from June 2018 to December 2020. The studies involving human participants were reviewed and approved by the Ethical Committee of Renji Hospital. The participants provided their written informed consent by their parents to participate in this study. One hundred and forty pediatric patients received liver biopsies within 2 years after LT. The pediatric patients without 2D SWE data (*n* = 12) or serum liver function (*n* = 13), or unsuccessful 2D SWE examination (*n* = 4) were excluded from the study. Additionally, in a normal situation, measurement of LSM in the left lobe is inappropriate because it is affected by cardiac pulsation. However, in pediatric recipients, as the majority of the pediatric recipients have a body weight of <25 kg, donation of the left lateral lobe (segments II + III) is sufficient in most cases. Considering the potential differences in the shape and texture of different liver segments, for comparability of data, the pediatric patients with whole liver or right lobe transplantation (*n* = 13) were also excluded from the study (Figure 1). A total of 98 pediatric patients were recruited, including 86 subjects with left lateral lobe transplantation and 12 subjects with left lobe graft. The underlying disease included biliary atresia, cholestatic cirrhosis, cryptogenic cirrhosis, tyrosinemia, and glycogen storage disease. All 98 patients underwent serum liver function tests, conventional ultrasound examinations, and 2D SWE measurement. Serum liver function tests included alanine aminotransferase (ALT), aspartate aminotransferase (AST), total bilirubin (TB), direct total bilirubin (DB), alkaline phosphatase (ALP), and gamma-glutamyl transferase (γGT) levels. The time interval between serum liver function examination and liver biopsy was less than 1 week. The time interval between 2D SWE measurements and liver biopsy was no more than 2 days.

### Two-Dimensional Shear Wave Elastography Measurement

An Aixplorer scanner (Aixplorer; SuperSonic Imagine SA, Aix-en-Provence, France) was used with a curvilinear transducer (1–6 MHz) and a linear array transducer (4–15 MHz). Ultrasound examination was performed by one radiologist (G.L.H. with 10 years of experience in liver transplantation ultrasound and 4 years of experience in 2D SWE measurement). The time interval between 2D SWE measurement and liver biopsy was less than 2 days. The pediatric patients were fed with milk during the examination or sedated with oral chloral hydrate (0.5–1 mg/kg body weight) before the test to keep quiet if necessary. A high-frequency linear transducer SL15-4 (4–15 MHz) was used. The pediatric patient was maintained in the supine position, and the left lateral (or left) lobe of the graft liver was scanned. The SWE sampling frame was 2 cm × 2 cm and was put at 0.5 cm below the liver capsule to avoid the influence. A 10-mm-diameter region of interest was positioned in the center of the sample frame with a complete and homogeneous filling color map. The examination was considered successful when most of the area of the interest box (>90%) could be filled with homogeneous color and maintained at least one calm breath. When taking the measurement, major blood vessels and biliary ducts in the liver graft should be avoided. Five qualified data were obtained from each patient, and the median values (with their range) were calculated (expressed in kilopascals). The 2D SWE value was 3.5–4 kPa for healthy children and 7.5–8.5 kPa for post-transplant pediatric patients with normal liver function.

### Histological Examination

Ultrasound-guided liver biopsy was performed with an 18-gauge needle automatic biopsy gun, and the pediatric patient was in the supine position. The length of the liver specimen was at least 15 mm with six portal spaces. The liver tissue strips were routinely fixed in formalin and embedded in paraffin. Hematoxylin-eosin (HE) staining was used to find the histologic change. Two hepato-pathologists blinded to clinical data and 2D SWE results made the pathological diagnosis. Masson staining was used to stage liver graft fibrosis based on the Scheuer score system. Substantial was defined as S2 or more significant.

### Statistical Analysis

All data were first analyzed for normality of distribution using the Kolmogorov-Smirnov test of normality. Continuous normal distribution variables were expressed as mean ± standard deviation (SD). Non-normally distributed variables are represented by median (P25, P75) and range. Categorical variables are represented by counts and percentages. A receiver operating characteristic (ROC) curve was performed to determine the liver fibrosis stage. A frequency distribution was obtained for choosing the optimal cutoff values of 2D SWE to maximize the sum of sensitivity and specificity for different fibrosis thresholds. Optimal cutoff values to predict fibrosis stages were identified. Sensitivity, specificity, positive predictive values, negative predictive values, and accuracy were calculated. Statistical analysis was performed using software (SPSS, version 22.0; IBM, Armonk, NY, United States). The graph was performed by GraphPad Prism (Version 8.0, GraphPad Software, La Jolla, CA, United States). A *P*-value of < 0.05 was considered statistically significant.

## Results

### Participants’ Clinical Characteristics

Ninety-eight pediatric patients underwent liver graft biopsy 2 years after LT (Table 1). The median age at LT was 8 months (6–14 months); liver graft biopsy was 4-year-old (43–65.5 months). The median interval between liver graft biopsy and LT was 41 months. The pediatric patients were divided into six groups according to pathological diagnosis; these were normal (steady recovery), DILI (drug-induced liver injury), fibrosis, BPR (biopsy-proven rejection), BC (biliary complication), and VI [virus infection, including Epstein-Barr-Virus (EBV)/Cytomegalovirus (CMV) and HBV], respectively. The Liver Stiffness Measurements (LSM) by 2D-SWE increased in pediatric patients with liver graft cirrhosis, liver graft rejection, biliary complications, and EBV/CMV/HBV infection. Also, the median value was 17, 12.2, 18.7, and 10.3 kPa, respectively.

**TABLE 1:** Baseline characteristics of the pediatric recipients.

Feature	Case (%) or median [range]
Gender (male/female)	42/43 (49.4/50.6)
Underlying disease (Biliary atresia/cholestatic cirrhosis/cryptogenic cirrhosis/tyrosinemia/glycogen storage disease)	75/4/3/2/1 (88.2/4.7/3.5/2.4/1.2)
Age at LT (month)	8 (6,14) [5, 120]
Age at liver biopsy (month)	51 (43, 65.5) [30, 192]
The time internal between LT and biopsy (month)	40 (32.5, 48.5) [24, 123]
2D SWE measurement (kPa)	8.1 (7.4, 12) [6, 31.8]
Alanine aminotransferase (IU/L)	28 (16, 162) [10, 776]
Aspartate aminotransferase (IU/L)	51 (28, 160.5) [20, 1,339]
Gamma-glutamyl transpeptidase (IU/L)	31 (12, 184.5) [6, 811]
Alkaline phosphatase (IU/L)	284 (217.5, 436) [19, 1,293]
Total bilirubin (μmol/L)	7.3 (5.75, 14.2) [2.5, 308.4]
Direct bilirubin (μmol/L)	3.2 (2.2, 6.35) [0.5, 214.1]

Categorical variables are represented as number (percentages), and non-normally distributed variables are represented as median (P25, P75) [range].

### Correlation of Liver Stiffness Measurements and Liver Complication in Pediatric Receipt

**FIGURE 2:**
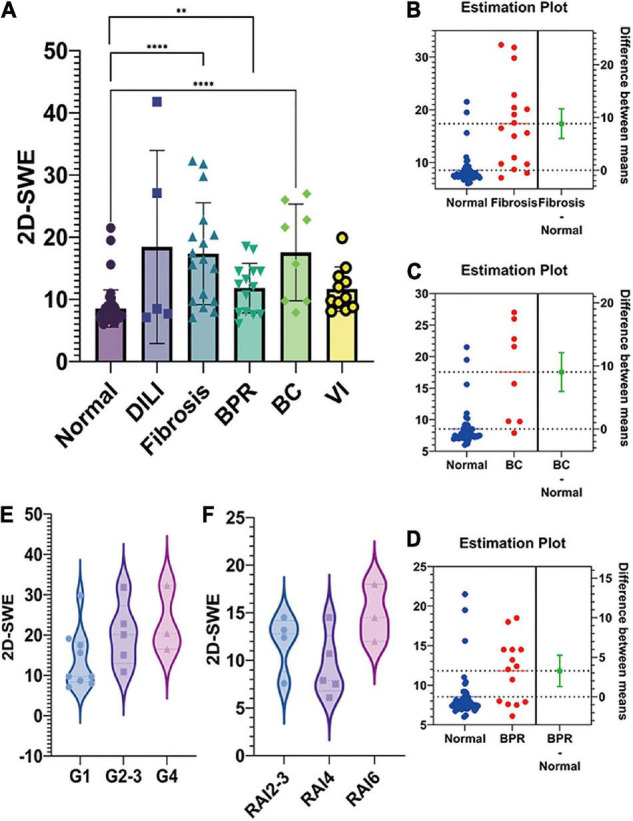
The liver stiffness measurement (LSM) and pathological findings of post-transplant pediatric liver biopsy. **(A)**. The LSM value of two-dimensional shear wave elastography (2D-SWE) was shown in the bar figure. 2D-SWE LSM value significantly elevated in post-transplant fibrosis, biliary complication, and biopsy-proven rejection (BPR). **(B)**. The difference between means of 2D-SWE LSM value was 8.824 ± 1.397 kPa IN FIBROSIS-NORMAL. The LSM value of patients with post-transplant biliary complication and biopsy-proven rejection also rose compared to normal recovery patients, with the difference between means at 9.025 ± 1.530 kPa **(C)** and 3.283 ± 0.9918 kPa **(D)**, respectively. **(E)**. The LSM ascended when the grade of fibrosis grew, despite no significant difference among each group (*p* = 0.166). This trend was not detected in acute rejection **(F)** (^*⁣*⁣**^p < 0.0001; ^**^p < 0.01).

The LSM value of 2D-SWE was significantly elevated in post-transplant fibrosis (*p* < 0.0001, Figure 2A), the difference between means was 8.824 ± 1.397 kPa (Figure 2B). The LSM value of patients with post-transplant biliary complication (*p* < 0.0001) and biopsy-proven rejection (BPR, *p* = 0.0016) also rose compared to regular recovery patients, with the difference between means at 9.025 ± 1.530 kPa (Figure 2C) and 3.283 ± 0.9918 kPa (Figure 2D), respectively. Additionally, the LSM ascended when the grade of fibrosis grew, despite there being no significant difference among each group (*p* = 0.166, Figure 2E). This trend was not detected in acute rejection (Figure 2F).

### Two-Dimensional Shear Wave Elastography and Pathologic Findings in Liver Graft Fibrosis

**FIGURE 3:**
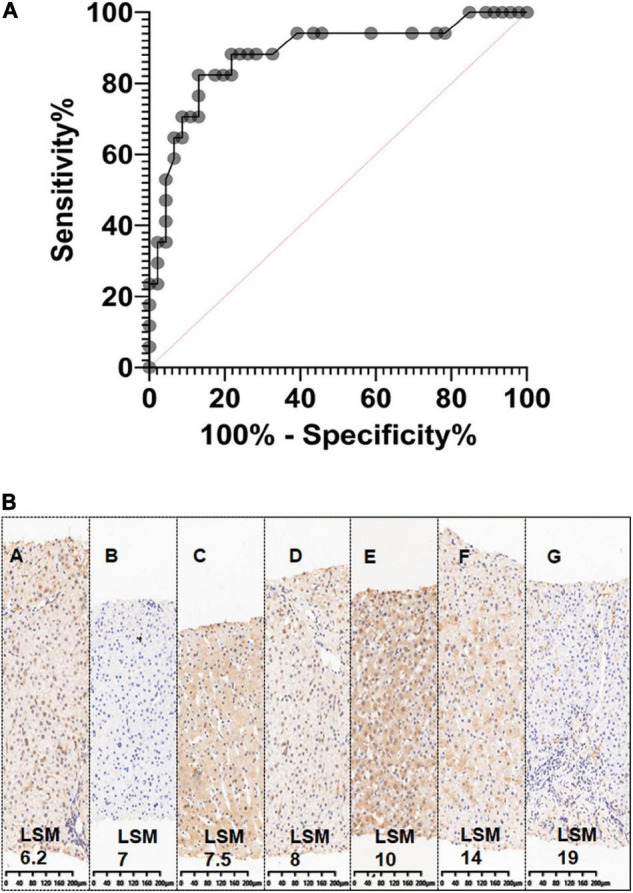
The sensitivity and specificity of two-dimensional shear wave elastography (2D-SWE). **(A)**. Graph shows receiver operating characteristic (ROC) for 2D SWE values in the liver graft to differentiate fibrosis and non-fibrosis. It also shows sensitivity and specificity of 2D-SWE in diagnosing liver graft fibrosis, the AUC was 88% (95% CI 0.78–0.99%); **(B)**. No correlation was detected between Liver Stiffness Measurements (LSM) and C4D staining (immunohistochemistry staining), a biomarker for antibody-mediated rejection (ABMR) induced tissue damage.

The 2D SWE value had a good diagnostic performance for predicting liver graft fibrosis, the AUC was 88% (95% CI 0.78–0.99%), the optimal cutoff value was 10.3 kPa, and the optimal cutoff value was 18.3 and 21.6 kPa for Grades 2-3 and Grade 4, respectively (Figure 3A). However, no correlation was detected between LSM and C4D staining, which is a biomarker for antibody-mediated rejection (ABMR)-induced tissue damage (Figure 3B).

**FIGURE 4:**
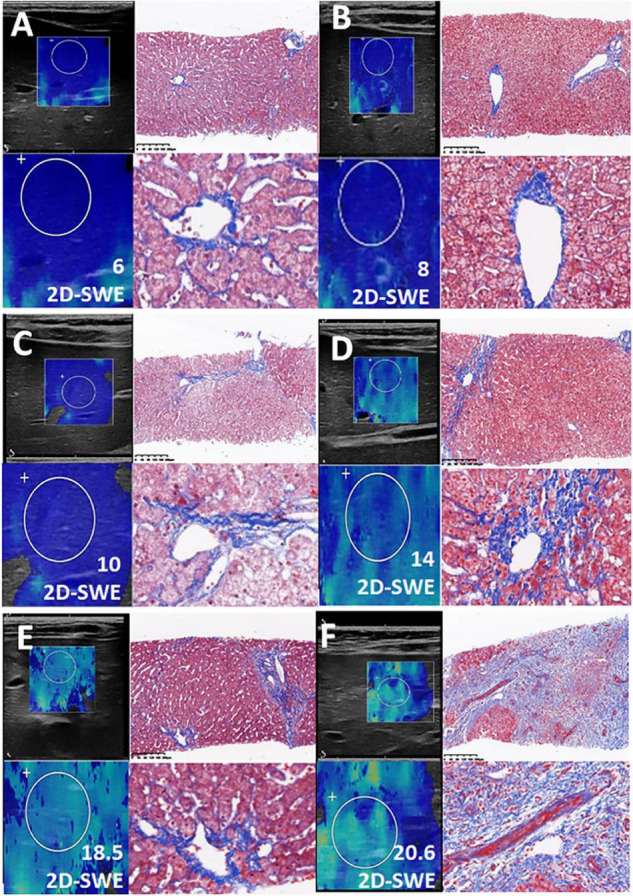
Consistent with the Liver Stiffness Measurements (LSM), the Masson staining of the biopsy was presented in this figure. **(A–F)** Liver fibrosis with increasing LSM value.

Although LSM also increased with elevated serum ALT levels (ALT ≤ 1ULN, 1ULN < ALT ≦ 2ULN, ALT > 2 ULN), the sensitivity and specificity of 2D SWE were still reassuring. For instance, in 44 pediatric patients with ALT ≦ 1ULN who had routine liver graft biopsy, Masson-staining was performed on 29 subjects. Scheuer’s score was graded as S0 in 10 subjects, S1 in nine recipients, S2 in seven recipients, and S3 in three recipients (Table 2). SWE could distinguish mild liver fibrosis (S1), liver fibrosis-S2, and significant liver fibrosis (S3) even in this situation. The AUCs were 0.68 (95% CI, 0.47–0.90) for ≥S1, 0.80 (95% CI, 0.63–0.97) for ≥S2, and 1 (95% CI, 1–1) for ≥S3, respectively (Table 3). The Masson staining of the biopsy was consistent with the LSM (Figure 4).

**TABLE 2:** Two-dimensional shear wave elastography (2D-SWE) values in the pediatric patients with normal serum ALT level (ALT ≦ 1ULN) after liver transplantation (LT) according to liver fibrosis stage.

Fibrosis stage	Number of patients	%	2D SWE value (kPa)
S0	10	34.48%	7.3 (6.6, 7.8) [6, 10.2]
S1	9	31.03%	7.4 (7.1, 7.6) [6.8, 8.5]
S2	7	24.14%	7.9 (7.4, 8.3) [7.1, 8.5]
S3	3	10.34%	14.5 (11.0, −) [11, 15.6]

Results are expressed as median (P25, P75) [range].

**TABLE 3:** Accuracy of two-dimensional shear wave elastography (2D-SWE) in predicting liver fibrosis in pediatric recipients with stable serum alanine aminotransferase (ALT) level (ALT ≦ 1 ULN) after liver transplantation (LT).

Fibrosis stage	AUC	Cut-off (kPa)	Sensitivity (%)	Specificity (%)	PPV (%)	NPV (%)	Accuracy (%)
**2D SWE**							
≥S1	0.68 (0.47-0.90)	7.35	73.68	60	77.78	54.55	68.97
≥S2	0.80 (0.63-0.97)	7.80	70.0	84.21	70.0	84.21	79.31
≥S3	1.00 (1.00-1.00)	10.60	100	100	100	100	100

2D SWE, two dimensional shear wave elastography; LT, liver transplantation; AUC, the area under the ROC curve; PPV, positive prediction value; NPV, negative predictive value.

## Discussions

Two-dimensional shear wave elastography is a newer elastography method using several lines of acoustic radiation force impulse to generate shear waves over a large region of interest within the liver in real-time to obtain quantitative images of the elasticity. 2D SWE can detect the propagation of the shear wave, obtain elastic images based on conventional ultrasonic images, and quantitatively measure liver stiffness (16). The successful rate of LSM by 2D SWE was above 98% in children (17, 18). The main factor affecting pediatric patient measurement was poor image quality caused by crying and movement, which can be solved by feeding and sedation. It has been reported that the LSM of the left lobe is higher than the right lobe, and the stability of the left lobe is less than the right due to the influence of cardiac pulse and abdominal vessel pulse (19, 20). In our study, LSM were all performed in left lobe liver graft, and most pediatric patients acquired successful value.

Liver stiffness measurements increased in pediatric patients with liver graft cirrhosis, liver graft rejection, drug-induced liver injury, EBV/CMV infection, bile duct complication, hepatic venous-occlusive disease, and hepatitis. Due to the liver capsule around the liver, the size of the liver is relatively fixed; when inflammation, edema, congestion, and cholestasis make the liver volume increase, the intrahepatic pressure rises. In addition, literature reported that patients with liver cholestasis, acute inflammation, bile duct complication, and hepatic vein obstruction lead to an elevated LSM, which is consistent with our findings (21, 22).

Recently, the pilot study reported by Vo et al. (23) has indicated that the LSM value of 2D-SWE was significantly elevated in pediatric LT recipients with liver graft fibrosis. However, 81.8% of patients’ graft type was whole liver, and the graft should be from Donation after Cardiac Death (DCD) or Donation after Brain Death (DBD). In this study, living donor liver transplantation (LDLT) with the left lateral segment (LLS) is the majority. Given the small sample size, Vo et al..could not reliably examine the impact of concurrent acute liver rejection and/or hepatic inflammation. The present study further demonstrated the role of LSM in the assessment of pediatric liver graft fibrosis based on a larger sample size from a more homogenous LT protocol. Considering abnormal ALT levels will affect LSM, in this study, pediatric patients were divided into three groups: ALT ≦ 1 ULN, 1 ULN<, ALT ≦ 2 ULN, and ALT > 2 ULN group. Zeng et al. (24) reported that remarkably elevated ALT levels (five times greater than normal liver function level) would elevate liver stiffness. Zeng et al. (25) distinguished double diagnostic thresholds in diagnosing liver fibrosis according to ALT level (ALT ≦ 2 ULN and ALT > 2ULN), thus, reducing the influence of ALT level on the evaluation of liver fibrosis.

In our study, the pathologic findings in thirteen patients were liver graft rejection, LSM was 7.8, 10.7, and 12.6 kPa in ALT ≦ 1 ULN, 1 ULN < ALT≦2 ULN, and ALT > 2 ULN groups, respectively. LSM increased with the elevated serum ALT level. In the patients with liver graft rejection but with normal serum ALT levels, LSM was not significantly increased. Yoon et al. (26) performed 2D SWE to monitor graft liver stiffness during the early postoperative period in adult LT; 2D SWE measurement could suggest graft liver rejection 4 weeks after LT in adult patients.

Scheenstra et al. (27) found that the incidence of fibrosis in pediatric patients after LT was extremely high, 34, 48, 65, and 69% at 1, 3, 5, and 10 years after LT, respectively. Evans et al. (28) found in 158 pediatric patients, with stable serum liver function levels after LT, that the incidence of liver fibrosis was as high as 17.7, 45.9, and 65.6% at 1, 5, and 10 years after LT, respectively. Venturi et al. (29) reported that graft fibrosis in pediatric patients was up to 74% 10 years after LT, 70% of which showed the liver function level was stable. Acute and chronic rejection, drug injury, viral infection, and vascular or biliary tract complications could cause liver damage and inflammatory response after LT. When the repair was excessive or out of control, abnormal hyperplasia and the deposition of the extracellular matrix formed liver fibrosis and gradually developed into cirrhosis (30). Thus, even in the pediatric recipients with stable liver function levels, the damage to the graft liver potentially happened.

In this study, we analyzed the pediatric recipients who received liver graft biopsy with normal ALT levels after LT; the pathological results showed liver fibrosis stages ≥S1, ≥S2, and ≥S3 were accounted for 65.5% (19/29), 34.5% (10/29), and 10.3% (3/29), respectively. LSM was satisfactory in diagnosing significant liver graft fibrosis with a sensitivity and specificity of 100%. However, the sensitivity and specificity for diagnosing mild liver graft fibrosis and significant liver graft fibrosis were 73.7 and 60%, 70 and 84.2%, respectively. Zhuang et al. (22) performed 2D SWE to assess liver fibrosis caused by chronic hepatitis B in an adult patient, and the sensitivity and specificity for significant liver fibrosis, severe liver fibrosis, and cirrhosis reached 92, 91.6, and 94.6% and 90, 96.7, and 94.9% respectively. The 2D SWE could also be performed by another platform, such as the LOGIQ E9 system (GE Medical Systems, Wisconsin, United States) (31).

### Limitations

Firstly, the enrolled children may not widely represent the pediatric recipients. In recent years, a routine liver biopsy was performed in our center, and the parents were not preferred for the invasive and possible complications after liver graft biopsy. Secondly, the pediatric recipient was not strictly fasting when taking the SWE examination, and the measuring was started while feeding (32, 33). Thirdly, we enrolled the pediatric recipients 2 years after LT to reduce liver function fluctuation. However, elevated liver function levels might still affect the cutoff value of liver fibrosis staging (34). Fourth, LSM by 2D SWE was performed in the left liver graft; literature reported that the stability and accuracy of LSM in the right lobe is higher than left lobe; in this study, pediatric patients have received a left lobe liver graft. Thus, we acquired satisfactory LSM in the left lobe.

## Conclusion

The LSM by 2D-SWE in the left liver graft was efficient. Besides liver graft fibrosis, elevated post-transplant pediatric LSM could also be present by liver graft rejection and biliary duct complications. Routine 2D-SWE evaluation could be optimal to predict significant liver graft fibrosis.

## Data Availability Statement

The original contributions presented in this study are included in the article/supplementary material, further inquiries can be directed to the corresponding authors.

## Ethics Statement

The studies involving human participants were reviewed and approved by Ethical Committee of Renji Hospital. Written informed consent to participate in this study was provided by the participants’ legal guardian/next of kin.

## Author Contributions

HFe, L-HG, and Z-FX: substantial contributions to conception and design, acquisition of data, analysis and interpretation of the data, and drafting of the manuscript. Y-CH, Z-CL, R-LG, HFa, and L-XJ: revising for critically important intellectual content. HFe and QX: final approval of the versions to be published. All authors contributed to the article and approved the submitted version.

## Conflict of interest

The authors declare that the research was conducted in the absence of any commercial or financial relationships that could be construed as a potential conflict of interest.

## Publisher’s note

All claims expressed in this article are solely those of the authors and do not necessarily represent those of their affiliated organizations, or those of the publisher, the editors and the reviewers. Any product that may be evaluated in this article, or claim that may be made by its manufacturer, is not guaranteed or endorsed by the publisher.

## References

[1] Kim WR Lake JR Smith JM Skeans MA Schladt DP Edwards EB OPTN/SRTR 2015 annual data report: liver. *Am J Transplant.* (2017) 17(Suppl 1):174–251. 10.1111/ajt.14126 28052604

[2] Feng S Demetris AJ Spain KM Kanaparthi S Burrell BE Ekong UD Five-year histological and serological follow-up of operationally tolerant pediatric liver transplant recipients enrolled in WISP-R. *Hepatology.* (2017) 65:647–60. 10.1002/hep.28681 27302659 PMC5159322

[3] Martinelli J Habes D Majed L Guettier C Gonzalès E Linglart A Long-term outcome of liver transplantation in childhood: a study of 20-year survivors. *Am J Transplant.* (2018) 18:1680–9. 10.1111/ajt.14626 29247469

[4] Kelly D Verkade HJ Rajanayagam J McKiernan P Mazariegos G Hübscher S. Late graft hepatitis and fibrosis in pediatric liver allograft recipients: current concepts and future developments. *Liver Transpl.* (2016) 22:1593–602. 10.1002/lt.24616 27543906

[5] Otte JB. Pediatric liver transplantation: personal perspectives on historical achievements and future challenges. *Liver Transpl.* (2016) 22:1284–94. 10.1002/lt.24470 27096329

[6] Bonis PA Friedman SL Kaplan MM. Is liver fibrosis reversible? *N Engl J Med.* (2001) 344:452–4. 10.1056/NEJM200102083440610 11172184

[7] Berchtold V Messner F Weissenbacher A Oberhuber R Entenmann A Aldrian D Influence of early biliary complications on survival rates after pediatric liver transplantation-A positive outlook. *Pediatr Transplant.* (2021) 25:e14075. 10.1111/petr.14075 34185384

[8] Schady DA Finegold MJ. Contemporary evaluation of the pediatric liver biopsy. *Gastroenterol Clin North Am.* (2017) 46:233–52. 10.1016/j.gtc.2017.01.013 28506363

[9] Dezsõfi A Knisely AS. Liver biopsy in children 2014: who, whom, what, when, where, why? *Clin Res Hepatol Gastroenterol.* (2014) 38:395–8. 10.1016/j.clinre.2014.05.002 24924903

[10] D’Onofrio M Martone E Brunelli S Faccioli N Zamboni G Zagni I Accuracy of ultrasound in the detection of liver fibrosis in chronic viral hepatitis. *Radiol Med.* (2005) 110:341–8. 16292241

[11] Dietrich CF Bamber J Berzigotti A Bota S Cantisani V Castera L EFSUMB guidelines and recommendations on the clinical use of liver ultrasound elastography, Update 2017 (Long Version). *Ultraschall Med.* (2017) 38:e16–47. 10.1055/s-0043-103952 28407655

[12] Bamber J Cosgrove D Dietrich CF Fromageau J Bojunga J Calliada F EFSUMB guidelines and recommendations on the clinical use of ultrasound elastography. Part 1: basic principles and technology. *Ultraschall Med.* (2013) 34:169–84. 10.1055/s-0033-1335205 23558397

[13] Cosgrove D Piscaglia F Bamber J Bojunga J Correas JM Gilja OH EFSUMB guidelines and recommendations on the clinical use of ultrasound elastography. Part 2: clinical applications. *Ultraschall Med.* (2013) 34:238–53. 10.1055/s-0033-1335375 23605169

[14] Aksakal M Oktar SO Sendur HN Esendaglı G Ozenirler S Cindoruk M Diagnostic performance of 2D shear wave elastography in predicting liver fibrosis in patients with chronic hepatitis B and C: a histopathological correlation study. *Abdom Radiol.* (2021) 46:3238–44. 10.1007/s00261-021-03019-6 33723676

[15] Furlan A Tublin ME Yu L Chopra KB Lippello A Behari J. Comparison of 2D shear wave elastography, transient elastography, and MR elastography for the diagnosis of fibrosis in patients with nonalcoholic fatty liver disease. *AJR Am J Roentgenol.* (2020) 214:W20–6. 10.2214/AJR.19.21267 31714842

[16] Ophir J Céspedes I Ponnekanti H Yazdi Y Li X. Elastography: a quantitative method for imaging the elasticity of biological tissues. *Ultrason Imaging.* (1991) 13:111–34. 10.1177/016173469101300201 1858217

[17] Gu LH Gu GX Fang H Xia Q Li FH. Shear wave elastography for evaluation of the urgency of liver transplantation in pediatric patients with biliary atresia. *Pediatr Transplant.* (2020) 24:e13815. 10.1111/petr.13815 32845544

[18] Franchi-Abella S Corno L Gonzales E Antoni G Fabre M Ducot B Feasibility and diagnostic accuracy of supersonic shear-wave elastography for the assessment of liver stiffness and liver fibrosis in children: a pilot study of 96 patients. *Radiology.* (2016) 278:554–62. 10.1148/radiol.2015142815 26305193

[19] Veiga ZST Perazzo H Fernandes FF Pereira GH Cavalcanti MG Peralta JM 2-D shear wave elastography for the evaluation of liver fibrosis in hepatosplenic schistosomiasis: reliability of a single measurement and inter-hepatic lobe variability. *Am J Trop Med Hyg.* (2020) 104:712–7. 10.4269/ajtmh.20-0032 33245042 PMC7866345

[20] Petzold G Grieme B Bremer SCB Knoop RF Goetze RG Ellenrieder V Prospective comparison of 2D-shearwave elastography in both liver lobes in healthy subjects and in patients with chronic liver disease. *Scand J Gastroenterol.* (2019) 54:1138–45. 10.1080/00365521.2019.1653961 31433262

[21] Piscaglia F Salvatore V Mulazzani L Cantisani V Schiavone C. Ultrasound shear wave elastography for liver disease. ultrasound shear wave elastography for liver disease. a critical appraisal of the many actors on the stage. *Ultraschall Med.* (2016) 37:1–5. 10.1055/s-0035-1567037 26871407

[22] Zhuang Y Ding H Zhang Y Sun H Xu C Wang W. Two-dimensional shear-wave elastography performance in the non-invasive evaluation of liver fibrosis in patients with chronic hepatitis b: comparison with serum fibrosis indexes. *Radiology.* (2017) 283:873–82. 10.1148/radiol.2016160131 27982760

[23] Vo HD Radio SJ Granader EJ Wojkiewicz LE Turner P Mauch TJ. Diagnostic performance of 2D-shear wave elastography and serum fibrosis markers for evaluation of hepatic graft fibrosis in pediatric liver-inclusive transplant recipients: a prospective pilot study. *Pediatr Transplant.* (2022) 26:e14225. 10.1111/petr.14225 35005824

[24] Zeng J Huang Z Jin J Zheng J Wu T Zheng R. Diagnostic accuracy of 2-D shear wave elastography for the non-invasive staging of liver fibrosis in patients with elevated alanine aminotransferase levels. *Ultrasound Med Biol.* (2018) 44:85–93. 10.1016/j.ultrasmedbio.2017.09.011 29122316

[25] Zeng J Zheng J Jin JY Mao YJ Guo HY Lu MD Shear wave elastography for liver fibrosis in chronic hepatitis B: adapting the cutoffs to alanine aminotransferase levels improves accuracy. *Eur Radiol.* (2019) 29:857–65. 10.1007/s00330-018-5621-x 30039224

[26] Yoon JH Lee JY Woo HS Yu MH Lee ES Joo I Shear wave elastography in the evaluation of rejection or recurrent hepatitis after liver transplantation. *Eur Radiol.* (2013) 23:1729–37. 10.1007/s00330-012-2748-z 23300037

[27] Scheenstra R Peeters PM Verkade HJ Gouw AS. Graft fibrosis after pediatric liver transplantation: ten years of follow-up. *Hepatology.* (2009) 49:880–6. 10.1002/hep.22686 19101912

[28] Evans HM Kelly DA McKiernan PJ Hübscher S. Progressive histological damage in liver allografts following pediatric liver transplantation. *Hepatology.* (2006) 43:1109–17. 10.1002/hep.21152 16628633

[29] Venturi C Sempoux C Quinones JA Bourdeaux C Hoyos SP Sokal E Dynamics of allograft fibrosis in pediatric liver transplantation. *Am J Transplant.* (2014) 14:1648–56. 10.1111/ajt.12740 24934832

[30] Zhou LY Jiang H Shan QY Chen D Lin XN Liu BX Liver stiffness measurements with supersonic shear wave elastography in the diagnosis of biliary atresia: a comparative study with grey-scale US. *Eur Radiol.* (2017) 27:3474–84. 10.1007/s00330-016-4710-y 28083694

[31] Akyuz M Gurcan Kaya N Esendagli G Dalgic B Ozhan Oktar S. The evaluation of the use of 2D shear-wave ultrasound elastography in differentiation of clinically insignificant and significant liver fibrosis in pediatric age group. *Abdom Radiol.* (2021) 46:1941–6. 10.1007/s00261-020-02844-5 33231728

[32] Horster S Mandel P Zachoval R Clevert DA. Comparing acoustic radiation force impulse imaging to transient elastography to assess liver stiffness in healthy volunteers with and without valsalva manoeuvre. *Clin Hemorheol Microcirc.* (2010) 46:159–68. 10.3233/CH-2010-1342 21135491

[33] Karlas T Pfrepper C Wiegand J Wittekind C Neuschulz M Mössner J Acoustic radiation force impulse imaging (ARFI) for non-invasive detection of liver fibrosis: examination standards and evaluation of interlobe differences in healthy subjects and chronic liver disease. *Scand J Gastroenterol.* (2011) 46:1458–67. 10.3109/00365521.2011.610004 21916815

[34] Zheng J Guo H Zeng J Huang Z Zheng B Ren J Two-dimensional shear-wave elastography and conventional US: the optimal evaluation of liver fibrosis and cirrhosis. *Radiology.* (2015) 275:290–300. 10.1148/radiol.14140828 25575116

